# Pathogen Inactivating Properties and Increased Sensitivity in Molecular Diagnostics by PAXgene, a Novel Non-Crosslinking Tissue Fixative

**DOI:** 10.1371/journal.pone.0151383

**Published:** 2016-03-14

**Authors:** Martina Loibner, Walter Buzina, Christian Viertler, Daniel Groelz, Anja Hausleitner, Gintare Siaulyte, Iris Kufferath, Bettina Kölli, Kurt Zatloukal

**Affiliations:** 1 Christian Doppler Laboratory for Biospecimen Research and Biobanking Technologies, Institute of Pathology, Medical University Graz, Graz, Austria; 2 Medical University Graz, Institute of Pathology, Graz, Austria; 3 Medical University Graz, Institute of Hygiene, Microbiology and Environmental Medicine, Graz, Austria; 4 Qiagen GmbH, Research and Development, Hilden, Germany; 5 LKH Hospital Weiz, Laboratory of Medical Department, Weiz, Austria; University of Torino, ITALY

## Abstract

**Background:**

Requirements on tissue fixatives are getting more demanding as molecular analysis becomes increasingly relevant for routine diagnostics. Buffered formaldehyde in pathology laboratories for tissue fixation is known to cause chemical modifications of biomolecules which affect molecular testing. A novel non-crosslinking tissue preservation technology, PAXgene Tissue (PAXgene), was developed to preserve the integrity of nucleic acids in a comparable way to cryopreservation and also to preserve morphological features comparable to those of formalin fixed samples.

**Methods:**

Because of the excellent preservation of biomolecules by PAXgene we investigated its pathogen inactivation ability and biosafety in comparison to formalin by in-vitro testing of bacteria, human relevant fungi and human cytomegalovirus (CMV). Guidelines for testing disinfectants served as reference for inactivation assays. Furthermore, we tested the properties of PAXgene for detection of pathogens by PCR based assays.

**Results:**

All microorganisms tested were similarly inactivated by PAXgene and formalin except Clostridium sporogenes, which remained viable in seven out of ten assays after PAXgene treatment and in three out of ten assays after formalin fixation. The findings suggest that similar biosafety measures can be applied for PAXgene and formalin fixed samples. Detection of pathogens in PCR-based diagnostics using two CMV assays resulted in a reduction of four to ten quantification cycles of PAXgene treated samples which is a remarkable increase of sensitivity.

**Conclusion:**

PAXgene fixation might be superior to formalin fixation when molecular diagnostics and highly sensitive detection of pathogens is required in parallel to morphology assessment.

## Introduction

For decades buffered formaldehyde solution (formalin) has been the gold standard for tissue preservation [1] in histopathological diagnostics [2]. Furthermore formalin is used for pathogen inactivation in vaccine production [3], and as an active component in disinfectants [4] underlining its favourable properties as a pathogen inactivating chemical. The development of nucleic acid-based molecular diagnostics has revealed several drawbacks of formalin fixation in molecular diagnostics, particularly in the context of personalized medicine. Formalin fixation leads to crosslinks between proteins and nucleic acids as well as to fragmentation [5] which adversely affects molecular analytical methods. Furthermore sequence artefacts arising from damaged DNA templates increase the risk of false-positive and false-negative calls in the diagnostic context [6]. Therefore the use of fresh or cryopreserved bio-samples is currently the preferred approach for optimal performance in molecular analyses. Conversely, collecting cryopreserved samples in routine health care faces several limitations. Due to the limited amount and size of human tissue samples available (e.g. biopsies), tissues cannot be processed in parallel by formalin fixation (required for histopathological diagnosis) and cryopreservation (for molecular diagnosis). Furthermore, cryopreservation cannot be applied as a routine procedure in health care for logistical and financial reasons. As a consequence a variety of alternative tissue preservation methods, such as alcohol-based fixatives UMFix (Sakura Finetek, Torrance, CA) [7], picrate fixative Bouin´s solution (Newcomer Supply, Middleton, WI) [5], HOPE [8] and RNAlater [9] were developed and tested as to whether they fulfil the required features of optimal preservation of tissue morphology and nucleic acids. Recently, the PAXgene Tissue System (PAXgene) (PreAnalytiX, Hombrechtikon, Switzerland) was developed by using a high throughput screening approach to find the best formulation for combined preservation of morphology and biomolecules [10, 11]. PAXgene is a commercially available non-crosslinking fixative comprising a fixation (PAXgene Fix) and stabilization solution (PAXgene Stab) based on a mixture of different alcohols, acetic acid and a soluble organic compound [10, 11]. Histological assessment of PAXgene-fixed paraffin-embedded (PFPE) tissues showed that morphological features were preserved comparable to formalin-fixed paraffin-embedded (FFPE) tissues [12–14]. Importantly, the preservation of nucleic acids in PFPE-tissues was shown to be of similar high quality as in fresh frozen samples [9–11]. Furthermore, proteomic analyses, such as Western blot and reverse phase protein arrays showed that detection of different proteins, including phosphor-proteins from human PFPE-samples was comparable to cryopreserved tissue [15, 16].

The evidence of well-preserved nucleic acids and proteins now raises the question whether PAXgene fixation results in proper inactivation of pathogens, or if other biosafety requirements have to be established for clinical personnel handling infectious human samples as is currently the case for formalin. Data obtained from immunocytochemistry assays showed that PAXgene inactivates influenza A virus, adenovirus and human cytomegalovirus (CMV) at least as well as formalin [17]. However, information on further microbiological species is lacking. Hence there is a major demand for analysis of the pathogen disabling properties of PAXgene compared to formalin. Therefore we tested the inactivation of 6 bacterial and 22 fungal strains. In addition we analysed the impact of fixation on CMV detection since it is highly seroprevalent [18], responsible for the most frequent complications after organ transplantations [19] and is the most important tissue-related viral indication for initiating pre-emptive therapy in organ transplant recipients [20].

Because of lack of specific guidelines for biosafety assessment of tissue fixatives, we followed the guidelines developed for accreditation of disinfectants (DGHM, German Society of Hygiene and Microbiology) [21, 22], the requirements for validation of sterilization procedures for bone transplants [23, 24] and CEN (European Committee for Standardisation) CT 216 EN 14485 for selecting test organisms for in vitro and cell culture assays. In all assays we compared PAXgene with formalin fixation for which long-term practical experience exists on biosafety risks, although this is rarely documented in the literature [25].

## Material and Methods

### Bacteria inactivation assays

To assess the inactivating property of PAXgene, reduction of colony forming units per millilitre (cfu/mL) was determined after fixation of different bacterial strains with PAXgene compared to formalin (4% formaldehyde buffered to pH 7.0). Phosphate-buffered saline (PBS)-treated bacteria served as reference. Reduction of bacterial growth of 10^5^ was considered as inactivated as this is requested for disinfectants [21, 22] and recommended for medical devices, blood products and bone transplants [23, 24].

*Clostridium sporogenes (Cs)*, *Staphylococcus aureus (Sa)*, *Bacillus subtilis (Bs)*, *Pseudomonas aeruginosa (Pa)*, *Mycobacterium smegmatis (Ms)* and *Mycobacterium terrae (Mt)* were obtained from ATCC or DSMZ (German Collection of Microorganisms and Cell Cultures) (Table 1).

**Table 1:** Investigated strains cover the spectrum of bacterial life conditions. Specific media were used for bacteria cultivation before and after inactivation treatment.

Bacterial strain	Spore forming	Growth condition	Liquid media (bioMerieux, France)	Solid media
*Clostridium sporogenes*, gram positive rod, DSM 1446	yes	anaerobic	Bouillon Schaedler + vitamin K3	Schaedler Agar + 5% sheep blood (bioMerieux, France)
*Staphylococcusaureus*, gram positive coccus, ATCC 29213	no	aerobic	Trypcase Soy broth	Columbia ANC Agar + 5% sheep blood (bioMerieux, France)
*Bacillus subtilis*, gram positive rod, DSM 347	yes	aerobic	Trypcase Soy broth	Chocolate Agar + PolyViteX (bioMerieux, France)
*Pseudomonas aeruginosa*, gram negative rod, ATCC 27853	no	aerobic	Trypcase Soy broth	Mc Conkey Agar (bioMerieux, France)
*Mycobacterium smegmatis*, gram positive rod DSM 43227	no	aerobic		CSA_Casein Soya bean digest agar; Blood agar + 5–10% human blood (Oxoid, England)
*Mycobacterium smegmatis*, gram positive rod, ATCC 359	no	aerobic		Middlebrook 7H10 Agar (Becton Dickenson, Germany)

**Fig 1:**
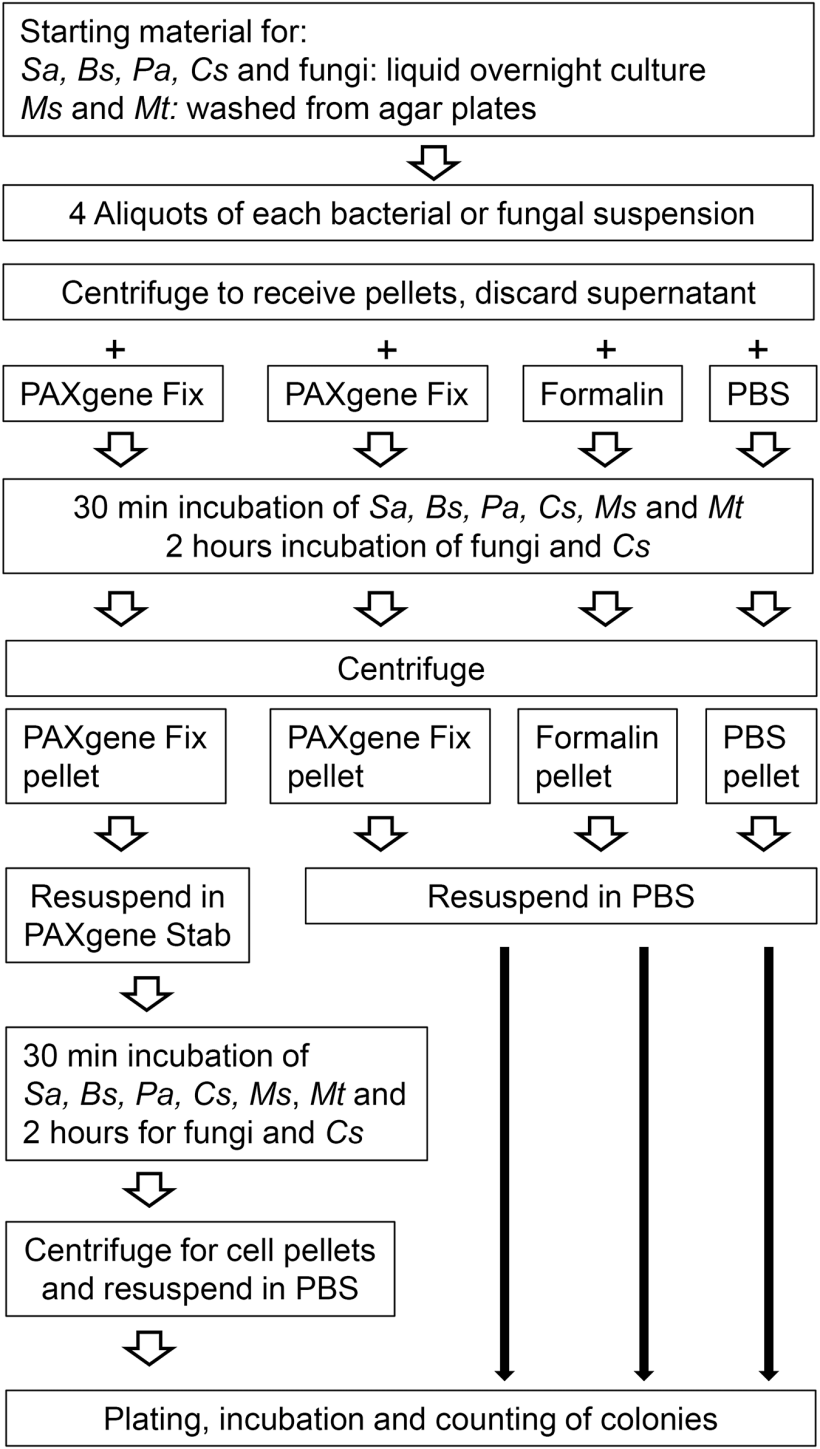
Work flow of bacterial and fungal inactivation experiments. Work flow of bacterial and fungal experiments. Bacteria experiments were performed with four (*Pa*) and six (*Ms*) independent experiments, respectively, when no colony was detected after inactivation, six experiments if colonies were detected (*Bs*, *Sa and Mt*) and seven experiments with the most variable strain *Cs*. Two-hour-treatments were performed with fungi (two experiments and all strains listed in Table 1) and additionally with *Cs* (three experiments).

Overnight cultures (ONCs) for *Sa*, *Bs* and *Pa* were prepared in appropriate liquid media (Table 1) with inoculation of a single colony and incubated overnight at 37°C and 200 rpm for aerobic conditions. For anaerobic conditions for cultivation of *Cs* ONCs were hermetically sealed and incubated at 37°C without shaking. After overnight cultivation 1 mL of the bacterial suspension was filled into 4 tubes per strain, centrifuged for 20 minutes at 1,200 x g and the supernatant was discarded. Mycobacteria strains were washed with PBS from a confluent cell layer of an agar plate. Due to extensive clotting of *Mt* cells were dissociated with the GentleMACS Dissociator (Miltenyi, Bergisch-Gladbach, Germany). One mL of each Mycobacteria suspension was filled into 4 tubes and centrifuged at 10,000 x g for 5 minutes. Each pellet was resuspended in 1 mL of the respective inactivation solution (Fig 1). Since the PAXgene procedure comprises two steps (i.e. PAXgene Fix followed by PAXgene Stab), both steps were tested separately and in combination. After 30 min incubation with fixatives or PBS at room temperature cells were centrifuged to pellets. One of two PAXgene fixed samples was resuspended in PBS, the second sample was incubated with 1 mL PAXgene Stab for another 30 min, centrifuged and resuspended in PBS. Dilution series of 1:10 were prepared and 100 μL were plated on the adequate agar medium (Table 1). *Bs*, *Sa* and *Pa* were cultivated under aerobic conditions at 37°C for 24–48 hours. *Cs* was anaerobically cultivated using the GENbag anaer system (bioMérieux, Marcy L’Etoile, France) at 37°C for 24–48 hours. *Ms* and *Mt* were incubated at 37°C for four and fifteen days, respectively. Six independent series of assays were performed with *Ba*, *Sa*, *Ms* and *Mt*, seven with *Cs* and four with *Pa*.

#### Sample processing and paraffin embedding

We investigated whether the paraffin embedding process following fixation in the context of histopathological analysis of tissue samples further inactivated fixation resistant bacteria. A standard embedding process comprised four ascending ethanol steps from 70% to 99% for four hours, followed by two hours isopropanol (Sigma-Aldrich, Steinheim, Germany), two hours xylene (J.T. Baker, Deventer, Netherlands) and three hours molten paraffin (ACM Herba-Chemosan, Vienna, Austria) at 55°C. ONCs of *Cs* were made as described above, incubated with 1 mL 70% ethanol for 30 min, centrifuged, resulting cell pellets were resuspended in 100 μL PBS, plated on appropriate agar plates and cultivated as described above. Since 70% alcohol fully inactivated *Cs* (no growth of colonies at any dilution) no further embedding steps were investigated.

### Fungi inactivation assay

Fungal strains (yeast and mould fungi) were obtained from culture collections ATCC, DSMZ, CBS (Fungal Biodiversity Centre) and patients’ isolates from the Biobank of the Medical University of Graz (Table 2).

**Table 2:** Human relevant fungi cultivated, treated with PAXgene and formalin and investigated for viability.

	Fungus / Group	Strain numbers
1	*Candida albicans /* Yeast	ATCC 90028, WB 005.09, WB 036.00
2	*Candida glabrata /Yeast*	DSMZ 11226, WB 015.09, B 011.02
3	*Candida parapsilosis /Yeast*	ATCC 22019, WB 005.01, WB 030.01
4	*Candida krusei /Yeast*	ATCC 6258, WB 022.03, WB 012.02
5	*Candida tropicalis /Yeast*	ATCC 90874, WB 004.04, 002.02
6	*Cryptococcus neoformans /Yeast*	ATCC 90112, WB 015.07, 011.05
7	*Geotrichum candidum /Yeast*	DSMZ 6401, WB 020.03, WB053.02
8	*Exophiala dermatitidis /Black Yeast*	CBS 207.35, WB 012.05, WB 028.11
9	*Aspergillus fumigatus /Mould*	ATCC 204305, WB 002.10, WB042.11
10	*Aspergillus flavus /Mould*	ATCC 204304, WB 038.11, WB011.03
11	*Aspergillus niger /Mould*	ATCC 16404, DSMZ 1988, WB032.08
12	*Aspergillus terreus /Mould*	DSMZ 826, WB 016.02
13	*Scedosporium apiospermum /Mould*	WB 002.12, WB 008.05, WB 017.08
14	*Fusarium solani /Mould*	WB 045.00, WB 030.11, WB 023.08
15	*Scopulariopsis brevicaulis /Mould*	WB 060.11, WB 007.05, WB 052.02
16	*Alternaria alternate /Mould*	CBS 109803, WB 004.06, WB 015.02
17	*Paecilomyces lilacinus /Mould*	WB 021.03, WB 034.00
18	*Penicillium chrysogenum /Mould*	WB 021.03, WB 034.00
19	*Rhizopus oryzae /Mould*	WB 012.06, WB 055.02, WB 027.08
20	*Rhizomucor pusillus /Mould*	WB 051.04
21	*Lichtheimia corymbifera /Mould*	WB 019.03, WB 037.11
22	*Cunninghamella bertholletiae /Mould*	WB 030.09, WB 014.03, WB 056.02

Cultivation and inactivation assays were performed according to the DGHM guidelines for disinfectants with modifications as follows. Four samples of yeast cells and spore suspensions respectively, with a turbidity equivalent to a McFarland 4 standard were prepared and centrifuged to cell pellets as described above for bacteria (Fig 1). Incubation with PAXgene and formalin was performed for 2 hours due to the higher resistance of spores. One hundred microliters of each of the fixed samples and dilution series of PBS control samples (mean 10^−12^) were plated onto Sabouraud agar plates (Oxoid, Basingstoke, UK) and incubated at 30°C for 48 hours. Two independent series of experiments were performed for each fungus strain.

### CMV inactivation assay

MRC-5 cells (human lung fibroblast cells, LGC Promochem, Germany, ATCC #CCL-171) were cultivated in 182.5 cm^2^ cell culture flasks (VWR, Vienna, Austria) with Minimum Essential Medium supplemented with GlutaMax (Gibco, Life Technologies, UK), 10% fetal calf serum (Gibco) and 1% Penstrep (Gibco) at 37°C and 5% CO^2^ until 60–70% confluency. Infection was performed with 2 mL suspension of human cytomegalovirus AD 169 (HPA #622, former Health Protection Agency, actually Public Health England, UK) containing 900 plaque forming units/mL per flask except negative control. Cells were cultured until massive cytopathic effects (CPEs) were observed (typically 10–14 days after infection). Cells were harvested using 0.05% Trypsin-EDTA (Gibco), centrifuged and resulting cell pellets were washed with PBS. Pellets were resuspended and distributed to 8 reaction tubes (1.5 mL). Two tubes each were incubated either with PBS (CMV-positive control), PAXgene Fix or formalin as described above for one hour. After removing PAXgene Fix by centrifugation, cells were stabilized for one hour with PAXgene Stab. One set of samples was centrifuged and cell pellets were washed with PBS, injected into 500 μL liquid 5% low melt agarose (Carl Roth GmbH, Karlsruhe, Germany) in a 1.5 mL reaction tube and immediately cooled on ice. Resulting agarose plugs were placed in tissue cassettes and processed in an automated tissue processor (Tissue Tek VIP, Miles Scientific, Sanova, Vienna, Austria). The second set of samples was not processed and paraffin embedded. All samples (paraffin-embedded and not paraffin-embedded) were dissociated in 5 mL MEM using a GentleMacs Dissociator (Miltenyi, Bergisch Gladbach, Germany). Floating paraffin was removed and cell lysates were applied to new MRC-5 monolayers grown in 75 cm^2^ cell culture flasks to detect viable virus. Cultivation was performed until CPEs appeared in PBS-treated cells (positive control). Cells were harvested on day 19 after infection with all lysates. To further investigate CMV viability on the basis of viral transcripts, RNA was isolated from all samples using AllPrep DNA/RNA/Protein Mini Kit (Qiagen). Quantification of these and all following extractions was performed on a NanoDrop 100 Spectrophotometer (PeqLab, Erlangen, Germany). Reverse transcription including DNAse-I-digestion was performed using QuantiTect Reverse Transcription Kit (Qiagen). Primers for immediate-early CMV gene *TRS1* (terminal right short 1) (NCBI Reference Sequence: NC_006273.2) were designed and blasted using NCBI primer design tool (www.ncbi.nlm.nih.gov/tools/primer-blast). Forward primer: acacagatggaacaaaagcaga; reverse primer: acgctgtggtttggagattga, amplicon (170 bp, Eurofins MWG Operon, Ebersberg, Germany). RT-qPCR was performed on Applied Biosystems 7900HT Fast Real Time PCR System (Applied Biosystems, Foster City, USA) using a TaqMan-specific set of PCR reagents following the manufacturer’s instructions. *Glyceraldehyde-3-phosphate-dehydrogenase (GAPDH)* was used as a reference gene. Forward primer: ccacatcgctcagacaccat, reverse primer: gtaaaccatgtagttgaggtc, amplicon (153 bp, Eurofins). Immunocytochemistry assays employing monoclonal mouse anti-CMV (M085401, Dako) were performed as described previously [17] confirming RT-qPCR results.

### Reverse transcription real-time PCR sensitivity assay

To investigate whether PAXgene fixation results in better sensitivity of PCR-based assays as compared to formalin fixation, MRC-5 cells were infected with CMV, harvested seven days post infection, centrifuged to obtain cell pellets and fixed either with PAXgene, formalin or PBS (as CMV-positive control) as described above. Three independent series with triplicate samples were performed. RNA of formalin fixed cells was isolated with RNeasy FFPE kit (Qiagen) without applying the deparaffination step at the beginning. RNA of PAXgene fixed cells was isolated using the PAXgene tissue RNA Kit (PreAnalytiX). For RNA isolation of PBS treated (CMV positive control) and not infected CMV-negative cells RNeasy Mini Kit (Qiagen) was used following manufacturer´s instructions. RNA quality for fixed samples was checked as previously reported [26]. To exclude that the observed differences in PCR sensitivity were due to different RNA isolation methods, RNA from an additional set of samples was isolated using the AllPrep DNA/RNA/Protein Mini Kit (Qiagen) for all fixation types. Reverse transcription was performed as described above. One hundred nanograms cDNA per tube were used in duplicates and three biological samples for RT-qPCR on a Rotor Gene Q 6000 Cycler (Qiagen) employing Rotor Gene SYBR Green PCR Kit (Qiagen).

### Quantitative real-time PCR sensitivity assay

MRC-5 cells were cultivated, infected with CMV, harvested two days after infection, pelleted, fixed either with PAXgene, formalin or treated with PBS (for control) as described above. Cell pellets were washed with PBS and homogenized with the GentleMacs Dissociator (Miltenyi) to release virus particles from the cells, viral DNA was isolated using the QIAamp MinElute Virus Spin Kit (Qiagen) and 20μl of template-DNA were used for detection of CMV performed with the IVD-approved artus CMV RG PCR kit CE (Qiagen) in duplicates according to manufacturer´s instructions.

#### Statistical analysis of PCR data

RT-qPCR and qPCR data on sensitivity (delivered from Rotor Gene Q Series Software 2.0.2, dynamic tube normalization) were analysed with IBM SPSS Statistics 22, Kolmogorov-Smirnov-Tests of Normality with Lillefors Significance Correction and T-test for paired samples (α = 0.05).

## Results

### Bacteria inactivation assay

Bacterial strains were treated with PAXgene and formalin according to the DGHM guidelines and European standards EN 1040 for bacteria, considering a reduction of more than 10^5^ for bacteria as sufficiently inactivated.

**Fig 2:**
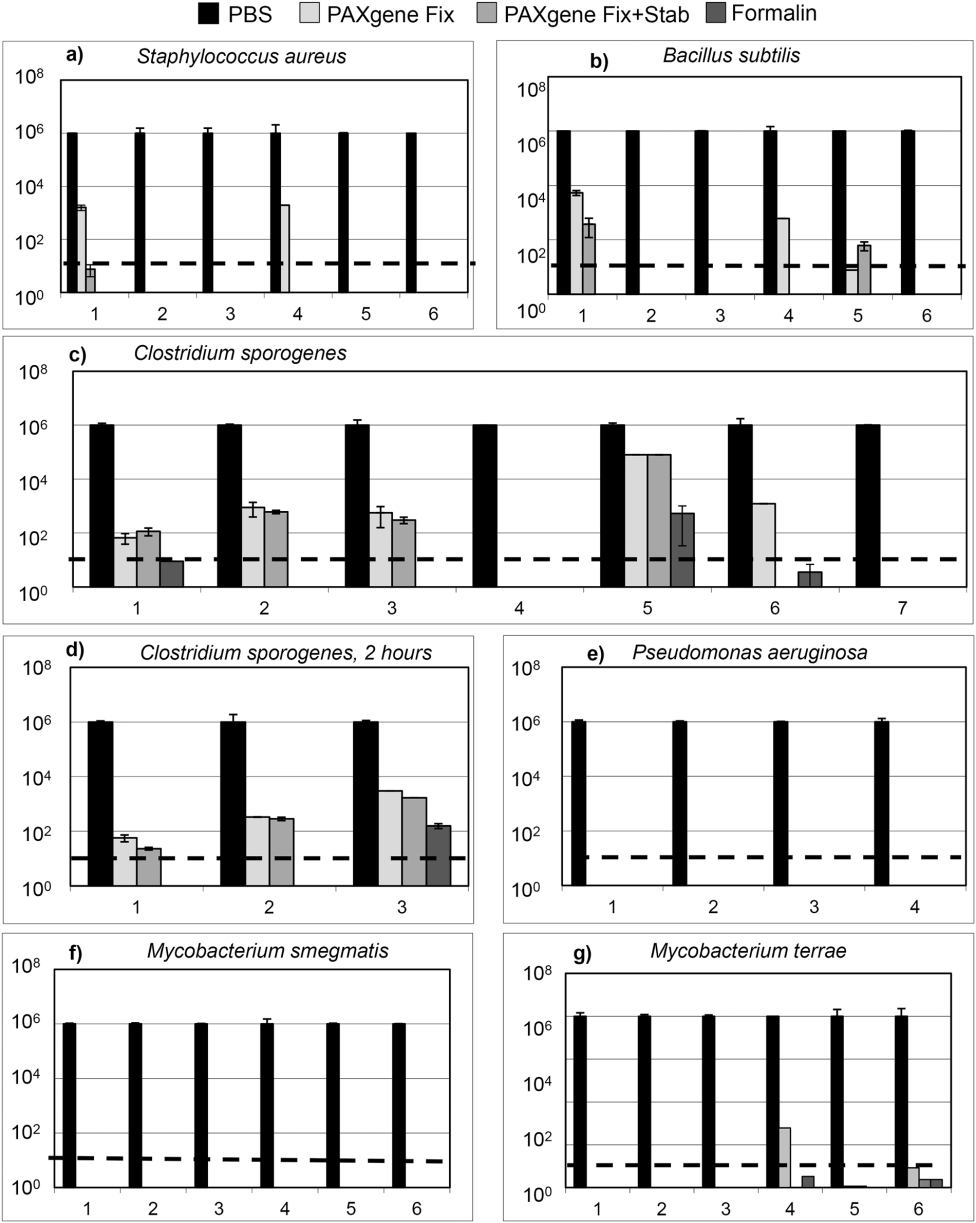
a,b,c,d,e,f,g: Results of bacterial inactivation experiments after treatment with PAXgene and formalin. Bacterial strains show variable viability after treatment with PAXgene Fix, PAXgene Fix and Stab compared to formalin and PBS (viability control) for 30 minutes (a, b, c, e, f, g) and 2 hours (d). The x-axis indicates the amount of experiments, the y-axis cfu/ml. To obtain comparable results all counted cfus were normalised to 10^6^. Columns represent the mean values (and error bars) calculated from the results of a series of different dilutions for one experiment. Dashed lines indicate the reduction limit of 10^5^.

*Sa* was inactivated by PAXgene (Fix and Stabilizer) in 6 out of 6 assays (Fig 2a). After treatment with PAXgene Fix only viability in 2 out of 6 assays was above the threshold. No colonies were detected after formalin treatment.

Inactivation of *Bs* was sufficient in 5 out of 6 series of assays for all fixatives (Fig 2b). Inactivation of less than 10^5^ was detected after PAXgene Fix treatment in 2 out of 6 assays as well as after treatment with PAXgene Fix and Stab in one assay.

No *Cs* colonies were found after 30 min PAXgene Fix and Stab in 3 out of 7 assays whereas 4 assays revealed different amounts of colonies (Fig 2c). No colonies or inactivation below the requested 10^5^ threshold was observed in 6 out of 7 experimental series with formalin, and in one assay the amount of colonies was not below the threshold. In an additional series of three assays with extended incubation time of 2 hours none of the PAXgene treated and only two of the series with formalin led to sufficient inactivation of *Cs* (Fig 2d).

Because *Cs* was the most resistant bacterial strain it was exposed to 70% ethanol, mimicking the starting condition of tissue processing to investigate synergistic inactivation effects of fixation and tissue processing. No colonies in any of the experiments were detected (data not shown).

*Pa* (Fig 2e) and *Ms* (Fig 2f) were the most sensitive bacterial strains and developed no colonies after fixation in any of four and six experimental series, respectively.

*Mt* colonies were detected after PAXgene treatment in three and after formalin treatment in two out of six series, but inactivation was more than log 5 in all six series.

### Inactivation of fungi

**Fig 3:**
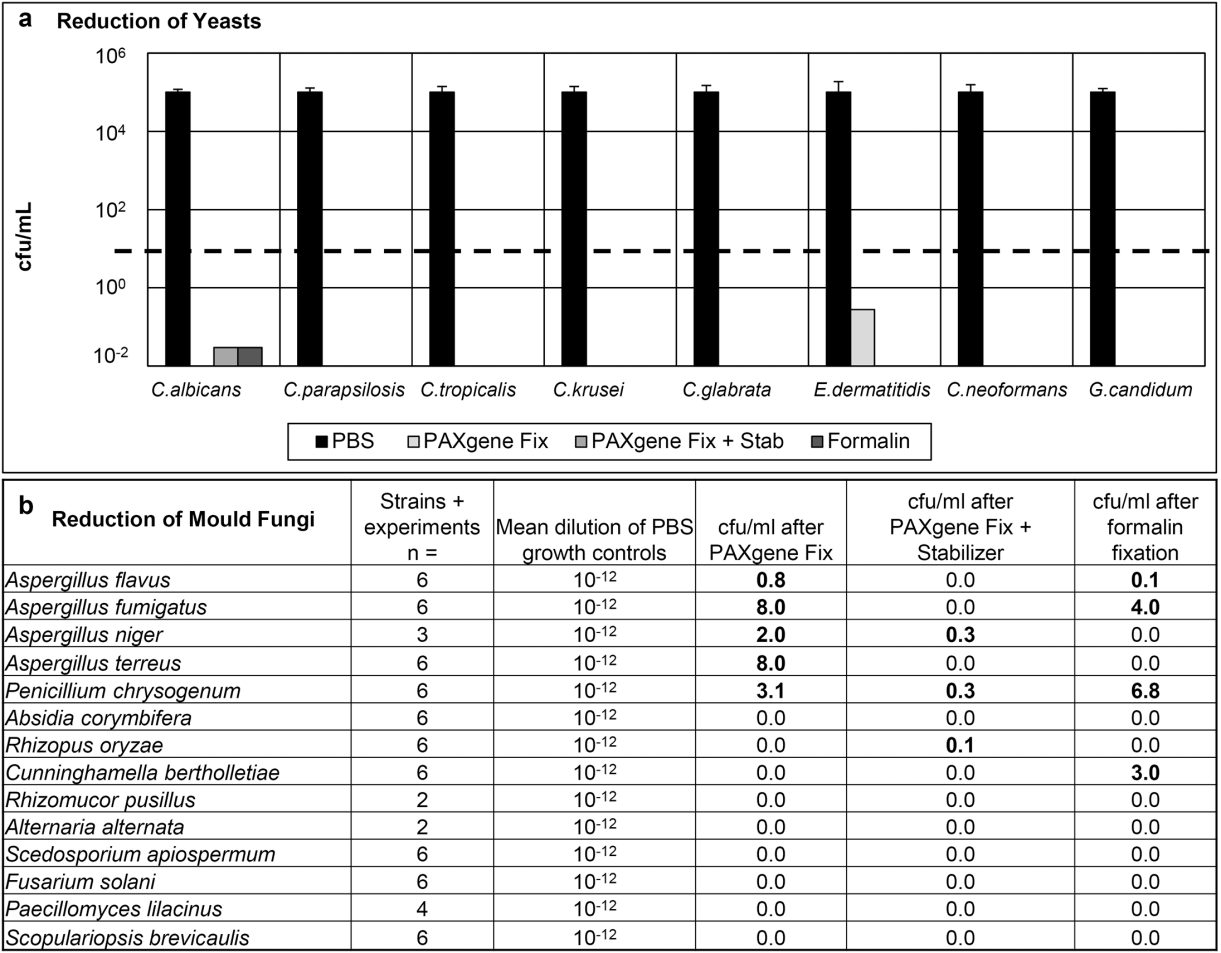
a,b: Results of inactivation experiments of human-relevant fungi by two hours fixation with PBS as positive control, PAXgene Fix, PAXgene Fix and Stab, and formalin. At least two assays per species (1–3 experiments) were performed. a) Cfu/mL was normalised to 10^5^. The dashed line indicates the threshold for minimum of reduction of 10^4^ used for disinfectants for fungi. b) Bold printed numbers indicate minimal growth after inactivation.

To expand the spectrum of human pathogenic microorganisms various species of fungi (Table 2) were used to investigate the inactivation ability of PAXgene compared to formalin. The starting concentration for all fungi inactivation assays was a turbidity equivalent to a McFarland 4 standard. According to DGHM guidelines and European standards EN 1275 (for yeasts) the requested reduction of more than 10^4^ cfu/mL for fungi was reached by PAXgene Fix alone, PAXgene Fix and Stab as well as by formalin for all fungal strains tested. With five different *Candida* species, some single colonies appeared after PAXgene (Fix and Stab) as well as after formalin treatment but the number was below the requested reduction threshold of 10^4^ (Fig 3a) in all assays. The most resistant species was the black yeast *Exophiala dermatitidis* showing most colonies and lowest reduction after PAXgene Fix only. PAXgene Fix plus Stab was as effective as formalin treatment. The yeasts *Cryptococcus neoformans* and *Geotrichum candidum* were inactivated with similar efficacy by PAXgene and formalin. For testing filamentous fungi (Fig 3b) mean dilutions up to 10^−12^ were necessary to receive evaluable numbers of colonies with PBS treated control samples. All four *Aspergillus* species and *Penicillium chrysogenum* yielded 0.8 to 8 cfu/mL after PAXgene fix treatment only. *Aspergillus niger*, *Penicillium chrysogenum* and *Rhizopus oryzae* showed 0.1 to 0.3 cfu/mL after PAXgene Fix and Stab. After formalin treatment, two *Aspergillus* strains, *Penicillium chrysogenum* and *Cunninghamella bertholletiae* 0.1 cfu/mL to 6.8 cfu/mL were detected.

### CMV inactivation

After PAXgene as well as after formalin fixation no specific CMV *TRS1* immediate-early gene transcripts were detected by RT-qPCR after 19 days of cultivation. Exposing CMV pellets to tissue processing conditions had no further effect because of complete inactivation already by fixation (data not shown).

### Impact of fixation on sensitivity of reverse-transcription real-time PCR assay

**Fig 4:**
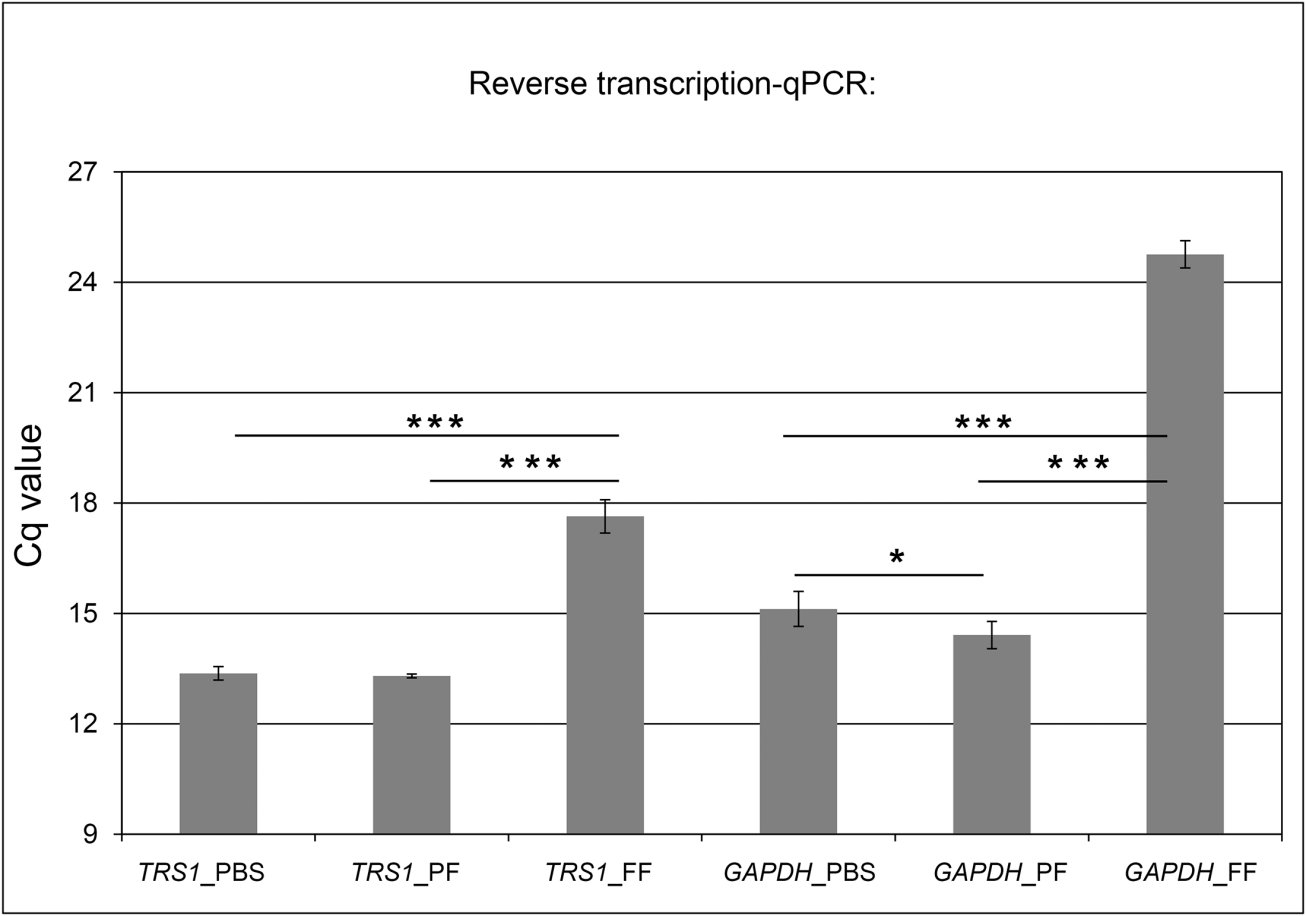
Reverse transcription qPCR: comparison of Cq values of PAXgene and formalin-fixed CMV samples. RT-qPCR sensitivity assay was performed to detect CMV early-immediate gene *TRS1* and reference gene *GAPDH* after fixation of CMV infected MRC-5 cells with PAXgene (*TRS1*_PF, *GAPDH*_PF), formalin (*TRS1*_FF, *GAPDH*_FF) and not fixed control samples (*TRS1*_PBS; *GAPDH*_PBS) in triple biological samples. Low Cq values indicate early detection. Statistical significance p < 0.0001 (***) or p < 0.03 (*).

Because PAXgene fixation led to markedly better preservation of RNA and DNA than formalin in human tissue we investigated whether these properties also increased the sensitivity of the detection of viral DNA and transcripts in biological samples, which might be beneficial for diagnostic applications. To address this question, CMV infected MRC-5 cells were fixed either with PAXgene (Fix and Stab) or formalin, and treated with PBS as control. RT-qPCR was performed to detect *TRS1* transcripts. Unfixed positive control (PBS) and PAXgene fixed samples resulted in essentially identical Cq (quantification cycle) values. After formalin fixation Cq values of *TRS1* were increased by a factor of 4 as compared to PAXgene, indicating a significantly higher sensitivity (p < 0.0001) after PAXgene fixation. This advantageous effect was even more pronounced for *GAPDH*, which was detected even 10 cycles earlier in PAXgene than in formalin fixed cells (Fig 4). The Cq value of no template control samples was higher than 31. In an attempt to exclude that these differences in PCR sensitivity were due to different RNA isolation methods used, RNA from all samples (PBS, PAXgene and formalin treated) was additionally isolated with the same isolation kit. However, the formalin as well as PAXgene treated samples were not suited for Allprep RNA isolation (Qiagen) which works well for PBS treated samples, making a direct comparison of isolation methods impossible (data not shown).

### Quantitative real-time PCR sensitivity assay

**Fig 5:**
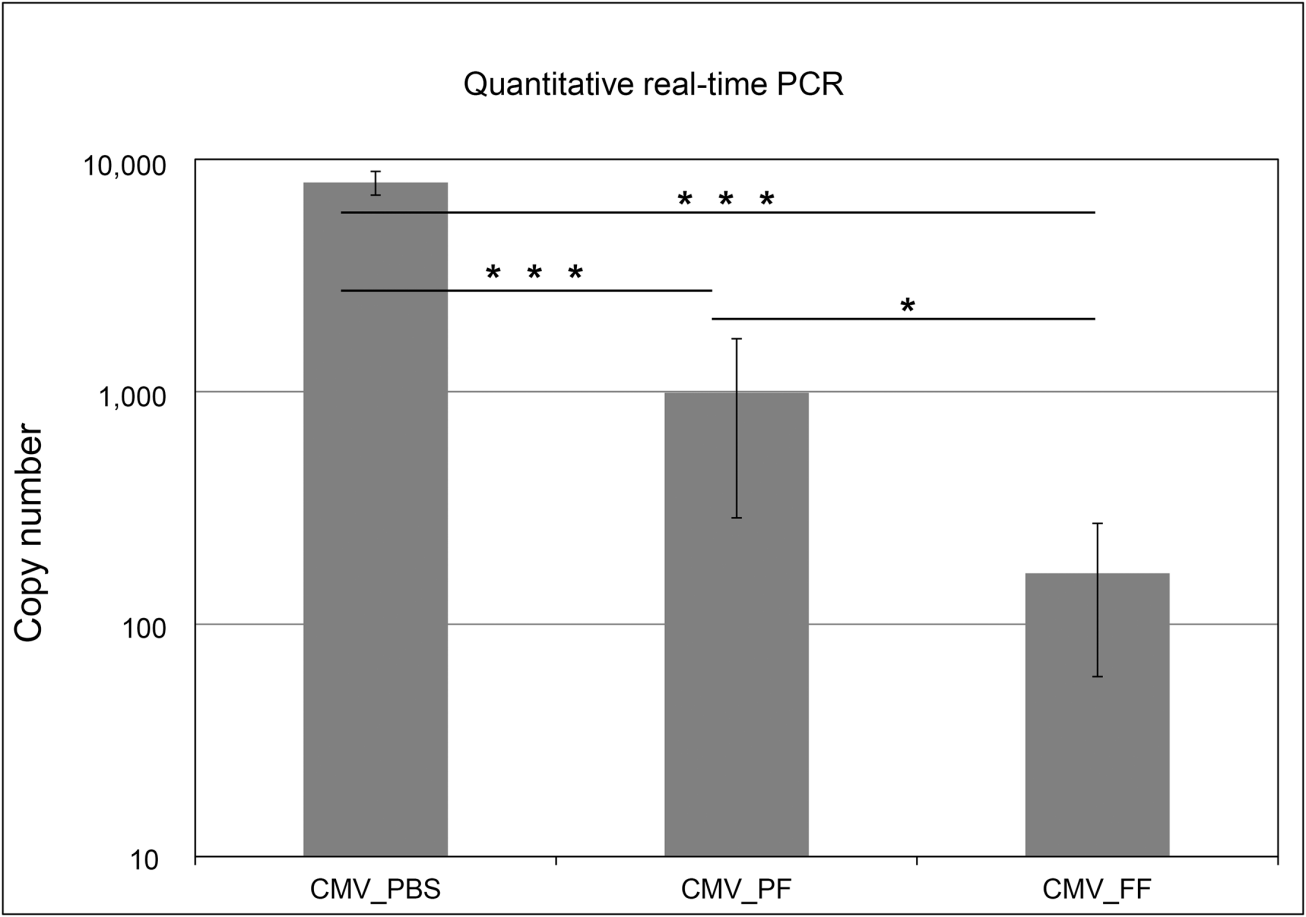
Quantitative real-time PCR, comparison of detected CMV copy numbers of PAXgene and formalin-fixed samples. CMV infected MRC-5 cells were cultured in triple biological samples. CMV-DNA copy numbers detected by IVD-approved artus CMV RG PCR Kit (Qiagen) show a significant difference between PAXgene (CMV_PF) and formalin fixed CMV (CMV_FF) infected samples compared to unfixed CMV samples (CMV_PBS). Statistical significance p < 0.0001 (***) or p < 0.05 (*).

To investigate the impact of PAXgene or formalin fixation on sensitivity of CMV DNA detection a quantitative real-time PCR assay employing the IVD-approved artus CMV RG PCR Kit (Qiagen) was used. CMV-DNA was isolated from infected MRC-5 cells as described above. The assay detected 8,000 copies/μL in PBS treated, 1,000 copies/μL in PAXgene and 165 copies/μL in formalin fixed samples. The difference between PBS and fixed samples (formalin and PAXgene) was highly significant (p < 0.0001) but also between PAXgene and formalin (p < 0.05). Earlier detection of CMV after PAXgene fixation compared to formalin is evident (Fig 5).

## Discussion

Fixatives that on the one hand preserve morphologic features well, and on the other hand do not modify biomolecules, are becoming increasingly important in the context of personalized medicine which often requires combined analysis of classical histo-pathological features and molecular biomarkers. The diagnosis of infectious diseases would also benefit from such fixatives which result in increased sensitivity in the detection of pathogens and, at the same time, provide the opportunity to correlate the presence of pathogens with morphological alterations in tissues. PAXgene, which has been developed and intensively evaluated in the context of the European Framework Programme 7-funded project SPIDIA (www.SPIDIA.eu), fulfilled both requirements. However, the exceptionally good preservation of biomolecules in PAXgene fixed tissues [9–11, 15, 16] raised concerns as to whether it sufficiently inactivates pathogens. Information on the inactivation capabilities of PAXgene were, therefore, required to decide whether PAXgene can be used following the same biosafety rules as established for health care workers involved in processing formalin fixed biological samples.

The results obtained in this study showed similar inactivation activity of PAXgene and formalin for bacteria and fungi. The relevance of the less efficient inactivation of *Cs* by PAXgene is difficult to interpret, particularly because only few systematic studies on formalin have been published, and due to the absence of specific guidelines for fixatives. Even formalin could not sufficiently inactivate *Cs* in three out of ten assays, independent of inactivation time suggesting incomplete inactivation capabilities. Reports on formalin concerning incomplete inactivation of picornaviruses causing poliomyelitis and foot-and-mouth disease [27] and a comparative study investigating different bacterial strains [25] are in line with our observation. Since routine tissue fixation is followed by tissue processing comprising exposure of infected samples to increasing concentrations of alcohol further inactivation of pathogens after processing and paraffin embedding is expected. Indeed, we found sufficient inactivation of the most resistant strain tested (*Cs*) by simulating the alcohol processing steps in our study. However there are also infectious agents, such as prions, which are not inactivated by formalin or alcohol [28]. Therefore, fixed tissues cannot be considered as not infectious in general and cautious handling following biosafety regulations is recommended independent of the fixation method [29].

Our studies with CMV not only confirmed the immunohistochemistry results of CMV inactivation by PAXgene as reported previously [17] by using a more sensitive RT-qPCR assay but also revealed potentially superior features of PAXgene fixation for molecular diagnosis of pathogens. There is an increasing need for more sensitive and accurate detection of pathogens, particularly in the field of transplantation medicine [30]. We found a 6-fold increased sensitivity for CMV DNA detection when employing an IVD-approved kit and a 16-fold significantly increased sensitivity to detect CMV transcripts in PAXgene fixed samples compared to formalin. Since a direct comparison of different fixatives in PCR-based assays is hampered by the fact that differently fixed samples may require different nucleic acid isolation protocols [26, 31] we compared the best achieved sensitivity using the optimised isolation protocol for each of the fixatives. PAXgene fixed samples more closely resembled unfixed samples in PCR-assays which are in line with previous observations that PAXgene interferes less with sample pre-analytics than formalin [10]. The properties of PAXgene fixation of excellent preservation of nucleic acids and morphology might be of particular relevance in the context of transplantation medicine where assessment of morphological features of organ rejection and highly sensitive molecular tests for detection of pathogens ideally have to be performed from the same tissue biopsies.
